# Achieving the Sustainable Development Goals: A Mixed Methods Study of Health-Related Water, Sanitation, and Hygiene (WASH) for Indigenous Shawi in the Peruvian Amazon

**DOI:** 10.3390/ijerph16132429

**Published:** 2019-07-08

**Authors:** Paola A. Torres-Slimming, Carlee Wright, Cesar P. Carcamo, Patricia J. Garcia, IHACC Research Team, Sherilee L. Harper

**Affiliations:** 1 Graduate´s School, Universidad Peruana Cayetano Heredia, Av. Honorio Delgado 430, Urbanización Ingeniería, San Martín de Porres, Lima 31, Peru; 2 School of Public Health, University of Alberta, 116 St & 85 Avenue, Edmonton, AB T6G 2R3, Canada; 3 Indigenous Health Adaptation to Climate Change, 116 St & 85 Avenue, Edmonton, AB T6G 2R3, Canada; 4 School of Public Health and Administration, Universidad Peruana Cayetano Heredia, Av. Honorio Delgado 430, Urbanización Ingeniería, San Martín de Porres, Lima 31, Peru

**Keywords:** indigenous health, Shawi, sustainable development goals, SDG-6, Peru, WASH, water security

## Abstract

Sustainable Development Goal 6 (SDG-6) addresses poor water quality, inadequate sanitation, and improper hygiene, all of which negatively impact health and disproportionately impact Indigenous Peoples’ health. Understanding and responding to local contexts is critical to effectively improve water, sanitation, and hygiene (WASH); however, in-depth understanding of local knowledge, practices, and perceptions are often overlooked. As such, this study described the knowledge, practices, and perceptions of WASH held by residents of two Indigenous Shawi communities in the Peruvian Amazon. Quantitative data were collected via a cross-sectional survey and analyzed using descriptive statistics. Qualitative data were collected via interviews, PhotoVoice, focus group discussions, and participatory transect walks, and analyzed using a constant comparative approach to thematic analysis. Emergent themes included characterizing water sources, collection methods, and consumption patterns; knowledge, perceptions, and practices related to WASH; and knowledge and perceptions of health issues related to WASH. This study provides insight into the ongoing challenges related to WASH in Indigenous communities in the Peruvian Amazon and highlights the need to prioritize interventions that will advance WASH-related SDGs.

## 1. Introduction

In 2015, the United Nations established the Sustainable Development Goals (SDGs), which are a set of 17 global targets designed to promote present and future prosperity for all by 2030 [1]. Of these goals, SDG-6 addresses issues of water scarcity, the appropriate use of water resources, and the improvement of human health by enabling local participation in water, sanitation, and hygiene (WASH) management [1,2]. Under the SDG targets for WASH for the upcoming decade, the World Health Organization and United Nations International Children’s Emergency Fund created a Joint Monitoring Program, which developed specific indicators to closely monitor progress related to SDG-6 [2]. The Joint Monitoring Program specifies that drinking water should be safe, affordable, and free from contamination [1,3]. In addition, appropriate sanitation practices should be promoted, with a particular focus on ending open defecation [4] and encouraging the use of soap in hand washing practices [5,6].

The SDGs prioritize vulnerable sub-populations [1,7,8], including Indigenous populations who are too often overlooked in international efforts to achieve the SDGs [9,10]. Achieving SDG-6 targets in these populations is particularly challenging considering historical and ongoing colonial practices [11,12], social exclusion, climate and environmental changes [13], and geographical isolation [14,15]. For example, Indigenous populations in the Peruvian Amazon experience disproportionate water security challenges compared to the Peruvian average [16]. In the Peruvian Amazon, less than 75% of the population has access to drinkable water from public networks, shared community taps, drilled wells or pumps, protected wells, rainwater, or other protected sources [17]; while only 5.5% of Indigenous communities have access to drinkable water through a public network [18]. Approximately 15% of Indigenous communities in the Amazon rely on well water supplies, whereas almost three-quarters of Indigenous communities collect untreated water from local rivers, ravines, or springs [18]. According to national data, approximately 20% of Peruvians practice open defecation [16,18]; however, this prevalence is higher in Indigenous communities given that less than 50% of Indigenous households have access to sanitation facilities, including latrines, pit latrines, or hanging toilets. As such, these communities typically experience a higher burden of water-related diseases compared to Peruvian national estimates [16,19]. These water- and sanitation-related challenges emphasize the systemic social, political, and environmental inequities that exist between Indigenous and non-Indigenous populations in Peru [7,13].

Despite a large base of evidence showing that adequate WASH practices are associated with better health outcomes, results from several studies [20,21] show that complex factors remain that influence individual, household, and community motivations to change behavior [21,22,23]. Consideration of the social, behavioral, and ecological factors that promote or inhibit appropriate WASH practices, such as the building and use of latrines, handwashing with soap, and the implementation of safe water practices, will lead to more effective WASH program outcomes [24,25].

A thorough understanding of the factors that affect attitudes, practices, and socio-cultural influences related to WASH in an Indigenous context is necessary in order to meet SDG-6 targets in the Peruvian Amazon. The aim of this study was to characterize water security in the context of the SDG targets for WASH in two Indigenous Shawi communities in the Peruvian Amazon. The study objectives were to examine and characterize: (i) water sources, collection methods, and consumption; (ii) knowledge, perceptions, and practices related to WASH; and (iii) knowledge and perceptions of the health issues related to WASH. This study advances our understanding of progress towards the SDG-6 targets for Shawi in Peru, and the results from this study provide broader insight into the importance of considering the inter-related and complex socio-cultural factors that influence water-related health in Indigenous communities. This greater knowledge base is intended to facilitate a more informed policy development process, with the ultimate goal of ensuring the right to water security for all Indigenous communities globally.

## 2. Materials and Methods

### 2.1. Research Approach

This study used an EcoHealth framework, which included principles of community participation, transdisciplinarity, systems thinking, social and gender equity, and knowledge-to-action throughout all stages of the research process [26]. For instance, the research project was co-developed in collaboration with members of the Shawi communities using transdisciplinary systems thinking approach—a research strategy that integrates two or more disciplines—to create a holistic framework (July 2015). Data collection involved a variety of participatory methods, with special consideration of social and gender equity (December 2015 and January 2016), and research results were interpreted and shared with the communities throughout the study. This research is part of a larger EcoHealth-guided initiative, the Indigenous Health Adaptation to Climate Change (IHACC) Program, which involves parallel studies in partnership with Indigenous peoples in Inuit Nunangat in Arctic Canada and Uganda (http://ihacc.ca/).

### 2.2. Study Location

The Shawi are one of the 65 ethnic groups in Peru. The Peruvian Shawi primarily live in the regions of Loreto and San Martin in the Peruvian Amazon, which collectively cover almost one-third of Peru’s landmass. These two regions are sparsely populated: Loreto has approximately 880,000 people with a population density of 9.6 pop./km^2^, whereas 380,000 people live in San Martin, corresponding to a population density of 2.5 pop./km^2^ [18,27]. According to the Ministry of Culture of Peru, there are 25,460 Shawi living in these regions [28].

**Figure 1:**
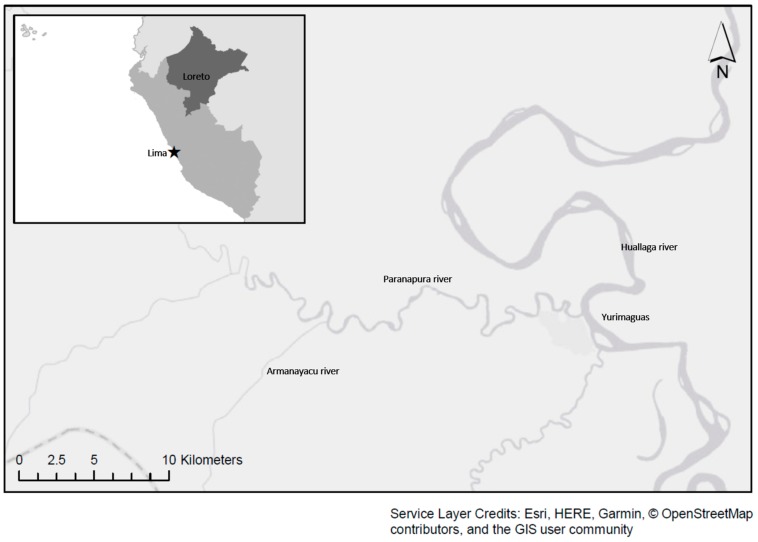
A map of Yurimaguas Armanayacu River and basin, Peru.

Two Shawi communities partnered in this study: Community A has a population of 68 people, and Community B has a population of 350 people. These communities are located on both sides of the Armanayacu River, which is a tributary of the Paranapura River that empties into the Huallaga River. Both communities are located in the Balsapuerto district within the Province of Alto Amazonas and the Region of Loreto in the Peruvian Amazon. Communities A and B are approximately twenty minutes apart by foot and are approximately two hours by motor vehicle from the nearest city, Yurimaguas (Loreto), depending on road conditions (Figure 1).

The Shawi have a very close cultural and spiritual relationship with nature and with river water in particular, and they rely upon the environment for subsistence. Their economy and livelihoods are based on hunting in the bush, small-scale agriculture, and fishing. They primarily drink *masato*, which is a sacred drink made from fermented *yucca*. Masato is prepared by women, who first boil the cassava, then beat or chew the mass until it is smooth. The mass is left to ferment for at least one week and is then mixed with water in a process called “*chapear*” before drinking [29].

### 2.3. Data Collection and Analysis

This study used a concurrent mixed methods approach, which involved a quantitative survey and qualitative PhotoVoice workshops, focus group discussions, interviews, and participatory transect walks. The quantitative and qualitative data were concurrently collected and analyzed.

### 2.4. Quantitative Data Collection

A cross-sectional survey was conducted in each community. The survey was designed to be a household-level census of the communities. One adult member per household completed one questionnaire. Respondents had to be older than 18 years and a resident in the community for at least the previous six months.

The questionnaire captured data on sociodemographic indicators as well as water security, hygiene, and sanitation information. All questions were revised and validated by Shawi researchers. The final version of the questionnaire was then translated from Spanish into the Shawi language. Three trained local Shawi research assistants administered the survey orally in the language preferred by the participant (i.e., Shawi language or Spanish) at the participant’s house.

### 2.5. Quantitative Data Analysis

The questionnaire responses were uploaded into mobile phones and automatically consolidated into a Magpi database (http://home.magpi.com/). When the questionnaire was administered in areas without internet access, the data were stored in the memory of the mobile phone and uploaded to the database upon arrival at the Universidad Peruana Cayetano Heredia in Lima. Data were then downloaded from the Magpi database and uploaded into Stata/SE version 15.0 (StataCorp, 4905 Lakeway Drive, College Station, TX, USA) for data cleaning and subsequent statistical analyses.

Descriptive statistics were performed for each independent variable to describe and summarize the data. Means of continuous variables were reported with their standard deviations. Categorical variables were compared using chi-squared or Fisher’s exact tests. For comparisons between continuous variables, a Student’s *t*-test was used. Variables with a *p* < 0.05 were reported as statistically significant.

### 2.6. Qualitative Data Collection

A variety of qualitative data collection tools and approaches were used to capture and triangulate the diverse understandings and lived experiences of water security in the two Shawi communities. Specifically, PhotoVoice, focus group discussions, in-depth interviews, and participatory transect walks were used to capture qualitative data.

Purposeful sampling was used for all qualitative methods, whereby particular groups with specific knowledge pertaining to the research question were sought for consultation and inclusion in the qualitative data collection process. The Principal Investigator in collaboration with the Local Researcher conducted the sampling process. Grounded theory was used as an inductive approach to data collection and analysis, which generated ecosystems theories that were grounded in data from the communities [30,31]. According to this approach, sampling, data collection, and analysis were conducted in iterations, whereby initial observations were used to direct subsequent methods, sampling targets, and analytical goals [32,33].

#### 2.6.1. PhotoVoice

PhotoVoice was used as a technique that equipped community members with cameras to take photographs in response to a research question and use the photographs as a foundation for group discussion and dialogue. This method has been utilized in public health promotion and research, providing resources so that people can identify, represent, and enhance their communities through photographic techniques [34]. This technique has been successful in engaging Indigenous communities in participatory research in other environmental health studies [35]. In Peru, we held PhotoVoice workshops in one Shawi community (Community A) with female (*n* = 3) and male (*n* = 3) participants, as well as children (*n* = 1 boy; *n* = 1 girl). The first workshop involved training in camera use and photographic principles. Then, participants set out to take photographs that represented or illustrated the importance of water or water security in their community. A second workshop was held one week later to discuss the photographs. Participants were asked to select three of their own photographs that most accurately reflected their opinions on their community’s strengths and concerns regarding water security. Then, through a reflective process, participants were asked about the histories, stories, and/or symbolism behind their photographs, as well as any ideas and possible solutions pertaining to the issues presented in the photographs. Participants took a total of 202 photographs. The discussions were audio recorded, with permission, and totaled 228 min of recorded discussion. In the final PhotoVoice session, each participant selected images to be included in a small book. Messages were written in Spanish and Shawi alongside each photograph. This small book was left with the *Apu* (community leader) and used by the local school to disseminate information to the rest of the community.

#### 2.6.2. Focus Group Discussions

Qualitative data were also collected via focus group discussions in both communities, which involved dynamic facilitated conversations with groups of participants to explore water security in the context of their cultural background, reality, and lived experience. Considering the potential intersection between water and gender roles, and following EcoHealth principles, one focus group discussion was conducted for each gender group in each community (*n* = 7 women and *n* = 8 men in Community A; *n* = 7 women and *n* = 11 men in Community B). The focus group discussions were co-led by a Shawi research assistant and lasted an average of 36 min. Maps and diagrams were used in all sessions to stimulate discussions. The focus group discussions were audio recorded, with permission, and a total of 218 min of discussion were documented.

#### 2.6.3. In-Depth Interviews

Semi-structured in-depth interviews were conducted with key informants in a conversational format to obtain key information about water security and safety, while also giving the participant freedom to explore new related topics spontaneously as the interview discussion unfolded. A total of four interviews were conducted with local individuals who were working in the area. The interviews were co-conducted with a local Shawi research assistant. The interviews were audio recorded, with permission, and averaged 60 min in duration. A total of 190 min of conversation were recorded.

#### 2.6.4. Transect Walking Paths

In this data collection activity, we walked with participants along a path to explore Shawi connections to their environment, their sense of place, and their relationships with water [36,37]. Participants described various local water resources, water security and safety problems, and opportunities that they faced in their daily lives. A total of eleven walking paths were audio recorded and photographed, with permission, with a total of 214 min of recorded discussions and 216 photographs taken.

### 2.7. Qualitative Analysis

A modified grounded theory approach was used in the qualitative data analysis [38], and involved thematic analysis [39] and a constant comparative method [40] via four iterative steps: (i) data familiarization; (ii) initial coding; (iii) identifying and developing themes; and (iv) reviewing, defining, and refining themes. First, audio recordings from each qualitative data collection method were transcribed verbatim, hand-checked for accuracy, and, if necessary, translated from Shawi to Spanish. To become more familiar with the data, the audio recordings were played while reading and re-reading the transcripts. Second, initial codes were generated using a hybrid inductive and deductive approach, whereby initial codes were theory-driven and subsequent codes were added and modified based on the data as they were collected [41]. Themes were developed by defining, refining, redefining, grouping, collapsing, and expanding codes into salient categories. This theme development process necessitated a constant comparison of ideas, concepts, and codes between and within interviews, demographic groups, and communities. This process was facilitated by reflective memoing, concept mapping [42], team debriefing sessions [43] and the development of a theme and codebook [44]. Finally, the validity of the developed themes was supported by data audit trails, team debriefing, member checking, triangulation, and prolonged immersion in the Amazon [43].

### 2.8. Ethical Considerations

This study followed ethical guidelines for research in Indigenous communities [45,46] and was approved by the Institutional Review Board of the Universidad Peruana Cayetano Heredia (approval number 65470), as well as Shawi community leaders. Prior to initiating the study, the *Apus* of both communities contributed to the development of the study proposal and agreed to collaborate throughout the project. Each study participant provided informed consent, and in the case of children, parental consent was obtained in addition to the assent of the child. For the PhotoVoice workshops, recruited participants were trained on the ethics of the study and the use of digital cameras to ensure that they understood the implications of doing photographic research.

## 3. Results

### 3.1. Household Demographics

A total of 73 occupied households were identified in the two communities. Eight of the households declined participation, yielding a participation rate of 89% (65/73). One additional household was excluded as questionnaire data were input incorrectly, leaving 64 households (12 in Community A and 52 in Community B) for the analysis. Regarding gender, 79.7% of the survey respondents were male. The average number of residents per house was 5.1 people, with an average of 2.3 adults and 2.8 children per house (Table 1).

The most common work activity in these communities was agriculture, which was practiced throughout the year (Table 1). Most households benefited from social programs. Of all respondents, 82.8% said they currently benefited from “*Programa Juntos*”, which is a conditional cash transfer program that was developed by the Peruvian Ministry of Development and Social Inclusion to promote access to health services and education for the poorest families in the country. In addition, 90.6% were registered for and using the National Healthcare Insurance “*Seguro Integral de Salud*” (SIS), which is provided by the Ministry of Health for Peruvians in poverty who do not have access to other health insurance systems. The Ministry of Health, alongside other organizations, also jointly administers the Child and Development Program (CRED), which provides periodic health evaluations and interventions (if necessary) to all children less than 5 years of age. The nearest health post that delivered the CRED program was located within forty minutes walking distance of the communities, and 79.7% of respondents reported using this program (Table 1). No participants were familiar with any deworming programs, including those delivered at the health post, in schools, or through health campaigns by the Peruvian Ministry of Health and/or non-governmental organizations (Table 1).

### 3.2. Water Sources, Collection Methods, and Consumption

**Figure 2:**
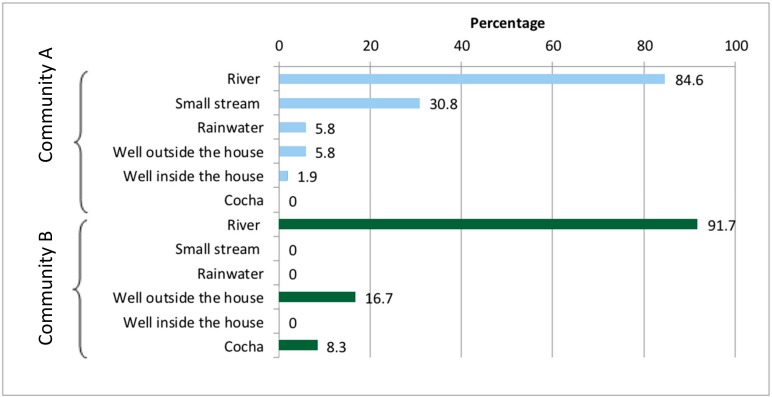
The water sources used for drinking, bathing, and washing, in each Shawi community in Yurimaguas, Armanayacu River and basin, Peru.

The majority of household survey respondents (85.9%) and interviewees indicated that their main water source was the Armanayacu River. Other sources of water included: water collected from small streams (25.0%); water collected from a well outside the house (7.81%); harvested rainwater (4.69%); and water gathered either from a *chocha* (i.e., pond) or a well inside the house (1.6% each) (Figure 2). Some families had built their own small wells with sedimentation systems, which were used for small daily activities such as cleaning dishes. If community members wanted to wash their clothes or bathe, they went directly to the river. There were four public wells in Community A which were built by foreigners, but they were not in use. The collected water was most often stored in buckets (98.4%), although 15.6% of respondents also reported that they stored water in bottles.

**Figure 3:**
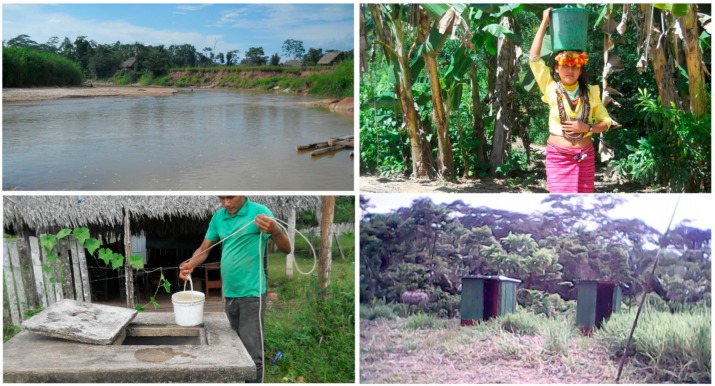
Photographs from transect walking paths in Shawi communities in Yurimaguas, Armanayacu River and basin, Peru. All photos taken and used with informed consent. Credits: Paola A. Torres-Slimming.

Water collection—from the Armanayacu river—was typically done very early in the morning and late in the afternoon (Figure 3). Most individuals carried either small 5 L or large 18 L buckets, and this was done four to five times per day to provide adequate water for daily activities, such as cleaning food and making *masato*. On average, it took community members 7–15 min to carry water from the water source into their homes. All family members, irrespective of gender or age, participated in bringing water to their homes.

Organized water collection occurred at local schools. The Parent’s Association or *Apu* delegated the responsibility of water collection to one family each day; that family then collected and carried water to the school in 18 L buckets. As one interviewee explained:

*Normally, mothers responsible for cooking bring water from the river. Every day they work, and every mother has her own commitment. I have a relationship with the mothers and they know who will take care of everyday responsibilities. They already know because I have pasted (the list) in the dining room so the moms can look at it*.

(I2)

The school in Community A also possessed a rainwater harvesting system. This water was used to prepare breakfast and lunch for students, and the food for these meals was provided through the Qali Warma National Schools Food Social Program.

To quench thirst, household survey respondents generally preferred drinking *masato* and reported that, on average, they drank 14.6 mocahuas (small clay bowls with an approximate capacity of 250 cc) of *masato* or water per day. Interviewees explained that they were accustomed to hydrating themselves primarily with *masato* from an early age. Purchased beverages were not common in either community, although when asked about purchased beverage preferences, 45.3% of households preferred buying soft drinks.

### 3.3. Knowledge, Perceptions, and Practices Related to WASH

Household survey respondents were asked about perceptions of the quality of their collected water (Armanayacu river), including taste, color, and smell. Using a Likert scale, 40.3% of household respondents indicated that the taste, color, and smell of collected water was “poor”. When asked about water for daily uses (e.g., washing, cooking, making *masato*), 38.1% stated it was of a “regular” quality. When asked about the acceptability of water from the river and small streams, 75.0% of individuals felt it was “drinkable”, 54.7% agreed that it can be used for bathing, and 56.3% considered the water to be “dirty” (Table 2). From those household survey respondents who considered the water source “dirty”, 58.33% considered the water “drinkable” versus 96.43% who considered the water source “not dirty” and “drinkable” (*p* < 0.001). Data not presented.

Community members felt that the river water was more turbid than it used to be, and it contained more sand than in previous years. Given this, individuals needed to let collected water rest for longer periods of time to allow the sediment to settle. Furthermore, people could no longer take midday baths in the river because they said the water was too hot. They attributed both of these phenomena to deforestation and a lack of trees near the riverbank, which caused the erosion of sand into the water, making it warmer. As one individual noted during a transect walk:

*First, you see dirty water with a lot of sand, and then, first thing you do when collecting the water is to pour the water into another container and let it settle. All the time is like that, when the children bathe, the sand gets up easily*.

(TWP6)

Regarding practices related to water purification, 59.4% of households reported heating or boiling water, 56.3% of households reported that they let the water stand to allow sedimentation in the storage container, and 3.1% added plants and/or herbs to the water (Table 3). No household reported purchasing water or using water filters or chlorine; however, over 80% of households were aware that chlorine is useful in preventing water-related illness. There were no significant differences in these water treatment practices between communities or genders (data not shown).

Regarding hand washing, 65.6% of households reported washing their hands before preparing *masato*; 68.8% before preparing their meals; 95.3% before eating; 73.4% after eating; and 35.9% after defecating (Table 3). Furthermore, 42.2% of household respondents stated that they “rarely” ran out of water for hand washing. There were no significant differences between communities or genders with respect to hand washing practices (data not shown). When asked about the use of soap, 59.4% of households stated that they washed their hands with soap when they possessed it (Table 3); however, interview participants explained that they rarely had access to soap.

All qualitative participants reported that they washed their hands before preparing the *masato* beverage, as they knew that this practice was good for their health; however, participants indicated that they only used water (with no soap) to rinse their hands before making *masato*, after cleaning food, or following open defecation. Participants explained that this was because they did not own soap and did not have access to a nearby store from which to purchase soap. Child PhotoVoice participants were especially conscious of hand washing practices, affirming they were taught about the importance of soap at their local school.

All households (100.0%) practiced open defecation; 37.5% of households reported defecating exclusively near their family home, and 50.0% openly defecated anywhere in the community (Table 3). As one transect walk participant explained, “*When we want to defecate, we dig a hole in the ground because we do not have a bathroom. We do not do it in the river* (TWP6).” Another participant reported: “*Sometimes children do defecate in the river, so we boil the water to drink; we have no other option: there is no other river—only when the river grows we take (water from) the stream* (TWP6).”

There were two latrines that were available for teachers and children at the local school. There were three other non-functioning latrines in the two communities. Most participants felt that river defecation was likely practiced by upstream communities and was responsible, in part, for the river contamination that they observed downstream. As one focus group discussion participant commented: “*The river is dirty and is contaminated with the feces from the residents from the upstream communities. That’s why a woman wants to make a well so that they can draw clean water from there* (FG1W).”

### 3.4. Knowledge and Perceptions of Health Issues Related to WASH

Respondents reported that their knowledge of hygiene practices predominantly came from outside the community, particularly from non-governmental organizations, evangelical groups, and/or health post practitioners. There were no local health workers or promoters in the communities to treat the symptoms of diarrheal illness (e.g., through oral rehydration or breastfeeding). However, interviewees mentioned that they would go to the health post for more severe diarrheal events, particularly if these cases occurred in children presenting with stomach aches, vomiting, diarrheal episodes with blood, and/or severe dehydration. Community members reported that they typically treated cases of diarrhea with their local healers or using their own family traditions. Some interviewees mentioned that diarrhea occurred due to drinking *masato* that was made with unsafe water, either because it was not boiled or was polluted. Some community members incorporated the knowledge and practice of boiling water, whereas others thought that water only needed to be heated (but not brought to a boil) to be safe for consumption.

In general, community members attributed increased episodes of diarrhea to river pollution. Qualitative analysis showed that participants believed this risk has increased over the past two decades due to more people throwing garbage (e.g., plastic bottles, cans, dead animals, and washcloths) into the river, in addition to an increasing number of individuals defecating directly into the river. Community members felt that upstream communities and foreigners were polluting the river water. As one focus group participant explained, “*I always say that the area of the River Armanayacu, that if people from upside river stream use it for washing, bathing, and defecating the downstream communities have to boil the water* (FG1M).”

## 4. Discussion

This study characterized knowledge, perceptions, and practices relating to WASH in two Shawi Indigenous communities along the Armanayacu River in the Peruvian Amazon. Results related to water sources, water collection methods, water-related practices, and knowledge and perceptions of health issues related to WASH highlighted the challenges in water access and sanitation faced by these remote populations. These results are an important starting point for visualizing opportunities for improved WASH practices and are intended to inform future policy planning to meet SDG-6 in the next decade.

For the Shawi communities in this study, untreated water was typically collected from the Armanayacu river. The quantity and quality of available water play an important role in the transmission of infectious diseases [47]. Some research suggests that water quantity can have a larger impact on health outcomes than water quality [6]. For instance, when a water source is located within 1 km of the home (e.g., a 30 minute round-trip), the availability of water closer to the home increases water consumption, sanitation, and good hygiene practices. This is known as the “water plateau” phenomenon [6]. Water collection took less than 30 min per round trip from the river, suggesting that the quantity of water available in these communities is sufficient for consumption, sanitation, and hygiene. Indeed, Shawi households reported that they rarely ran out of water, and collected 20-90 L of water per day per household [16,17,18].

Although the quantity of water available was not reported to be problematic in these communities, the Shawi reported that the quality of the water was an important concern, which is consistent with the statistics of limited water access, poor quality, and lack of cultural sustainability in Peruvian Indigenous populations [16,17,18]. Indeed, the river was not free from contamination, and water treatment (e.g., boiling and chlorination) was not commonly practiced. Relying on unprotected surface water sources can present important health challenges. For instance, past research has documented associations between unprotected and untreated surface water and water-related diseases, such as diarrheal disease and skin conditions [48,49]. Furthermore, water-related diseases can create and/or exacerbate existing undernutrition [50,51,52]. This is a particular concern for Indigenous populations, as these populations often experience a disproportionately high burden of disease in general as well as high levels of food insecurity and undernutrition [52].

Successful deworming programs along river basins have been reported in several countries [3,53] and World Health Organization (WHO) guidelines recommend that deworming programs should be mandatory in areas where the prevalence of any soil-transmitted infection is more than 20%, or where open defecation is practiced [3,54]. For the Shawi, open and river defecation were prevalent because latrines were not available to all community members. However, the Shawi reported that government and non-governmental deworming programs were not recently nor frequently offered at the schools or health posts. This inequity in WASH-related health service provision underscores the importance of the SDG concept ‘leave no one behind’, which focuses on addressing the inequalities experienced by vulnerable sub-populations, such as the Shawi [55,56,57].

Although most Shawi reported washing their hands with water, soap was often not available and therefore not commonly used in handwashing. However, handwashing with soap is an important SDG-6 target, as it is critical for public health. Indeed, a published systematic literature review found that only 19% of the world’s population used soap and water for hand washing after contact with feces [5,58]. Handwashing interventions are known to successfully decrease rates of diarrhea and the associated global burden of disease [59,60]. In an interventional study done by Curtis et al. in 2011, they found that handwashing with soap and water had better results in removing fecal bacteria from hands than handwashing with water alone, concluding that soap is an effective intervention for reducing diarrheal transmission [61].

Our research results identified several WASH challenges, including a lack of infrastructure resulting in open defecation and river defecation, sufficient water quantity but notable water safety concerns, and a lack of access to soap for handwashing. In the context of SDG targets, this places the Shawi in the bottom rung of the SDG-6 ladder [21,62,63]; given this, it will be important for Peru to prioritize WASH improvement efforts in Shawi communities—and other Indigenous communities in the Amazon—to meet their SDG targets, as these populations are well below national and international service provision targets [17,64]. In the context of the Transtheoretical Model (Stages of Change), Shawi were in the contemplation stage of change: people understood the health benefits of both using soap and latrines, but these resources were not available to support healthy behavior change.

It is well established that many sanitation programs are culturally irrelevant, locally inappropriate, and have inadequate reach throughout Africa and Latin America [65,66]. The Shawi communities in this study fit within this broader pattern. Indeed, although there were four foreign- built wells in one community, these wells were not functional. Furthermore, there were three closed latrines at the school; however, these were only available to teachers and children. To address these challenges, recent studies indicate that a better understanding of social and cultural norms play an important role in the adoption of new technologies and infrastructure [67,68]. As an example, the Canadian Government has taken steps towards strengthening regulatory frameworks into their WASH policies by incorporating aspects of cultural backgrounds for reducing water-related Indigenous health inequalities [69,70].

Our study found that more than 80% of community members benefited from a social program, highlighting an opportunity to integrate WASH initiatives into the existing food programs that were commonly accessed by the Shawi in these communities. A multi-sectoral approach that encompasses both water and food reflects a holistic Shawi cosmology and could have cascading effects that would move towards achieving several SDGs [19,71]. For instance, research has shown that the outcomes of programs designed to improve water quality, sanitation infrastructure, and hygiene behaviors improved when they were coordinated alongside critical health, nutrition, and food security interventions, particularly in communities with high rates of chronic malnutrition [72,73].

In the Shawi communities, there was organized water collection for the local schools; this presents an opportunity to integrate WASH education into the existing community water collection practice. Indeed, the importance of schools as a source for education in WASH promotion has been demonstrated in several studies, including improved knowledge of the importance of handwashing with soap resulting from health promotions at schools [74,75,76]. Integrating WASH education into the Shawi community school water collection program would not only reach schoolchildren, but also all household members who participate in this practice. This demonstrates the power of understanding local water culture and practices in order to make WASH initiatives more locally relevant and culturally appropriate, and ultimately more effective [77,78].

An important strength of this work was the combination of quantitative survey data with multiple forms of qualitative data. Additionally, the EcoHealth approach allowed us to explore the complexity of interrelationships between ecosystems and society through a community-based, participatory, transdisciplinary approach that considered gender, social equity, and sustainability. This approach helped to provide deeper insights into WASH practices and realities in Shawi communities in Peru by providing rich datasets with enhanced reliability and credibility through the triangulation of results. Although we found a convergence of results between the qualitative and quantitative data, it is important to consider that self-reported behaviors may be susceptible to social desirability bias. Furthermore, we conducted our study in two rural Shawi Indigenous communities in Balsapuerto, in the Region of Loreto in the Peruvian Amazon; considering the heterogeneity of Indigenous communities within the Peruvian Amazon, our results should be generalized to other Indigenous populations with caution.

## 5. Conclusions

Although the quantity of water available for these Shawi communities was sufficient, important water quality challenges persist. Open defecation and river defecation were widely practiced due to a lack of latrine infrastructure, and adequate handwashing was uncommon due to lack of both handwashing facilities and soap. These findings place Shawi communities, and likely other Indigenous communities in the Amazon, at the bottom of the ladder for SDG-6 indicators. Failed WASH initiatives from government and non-governmental organizations, as well as a lack of local community health promoters, make it difficult to implement sustainable improvements in WASH goals, and will challenge Peru’s ability to meet SDG-6 targets. We suggest that future research and public health initiatives should focus on understanding behaviors and norms within their communities before implementing WASH interventions and monitoring programs. Improved and locally relevant WASH initiatives will help reduce the gaps that persist and will allow the development of clear, community-oriented objectives for achieving SDG-6.

## Figures and Tables

**Table 1:** The description of sociodemographic variables in the two Shawi communities, Peru (*n* = 64).

Sociodemographic Variables of Households	*n*	(%)
Community A	52	(81.2)
Community B	12	(18.8)
Household respondent male	51	(79.7)
Number of residents per house *	5.1	(2.1)
Number of adults per house *	2.3	(0.9)
Mean number of children per house *	2.8	(1.8)
Social Assistance Programs	*n*	(%)
Comprehensive health insurance (SIS)	58	(90.6)
Children in the Growth and Development Program (CRED)	51	(79.7)
No deworming program	64	(100.0)
Government cash transfer assistance (JUNTOS)	53	(82.8)
Main household working activities	*n*	(%)
Agriculture as a full-time activity	63	(98.4)
Households with children participating in work	47	(73.4)

* Mean (standard deviation).

**Table 2:** The description of knowledge and perceptions towards water quality in the two Shawi communities, Peru (*n* = 64).

Knowledge and Perceptions towards Water Quality	*n*	(%)
Quality of water collected (taste, smell, color):		
“Poor”	25	(40.3)
“Regular”	14	(22.6)
“Good”	23	(37.1)
Quality of water for daily drinking (taste, smell, color):		
“Poor”	16	(25.4)
“Regular”	24	(38.1)
“Good”	23	(36.5)
Quality of water from the river and small streams:		
“Poor”	27	(43.5)
“Regular”	15	(24.2)
“Good”	20	(32.3)
Acceptability of water coming from the river and small streams:		
Water is drinkable	48	(75.0)
Water can be used for bathing	35	(54.7)
Water is dirty	36	(56.3)
Source of liquids that the household drinks:		
River water	16	(25.8)
Masato	46	(74.2)
Preferred drinking water source:		
River water	10	(16.1)
Masato	46	(74.2)
Soda	6	(9.7)
Average *mocahuas* † of *masato* or water per day *	14.6	(7.7)
Household prefers to purchase drinking water:		
No	60	(96.8)
Yes	2	(3.2)
Preferred purchased drinks:		
Water	2	(3.1)
Soda	29	(45.3)
Juice	7	(10.9)
Alcoholic drinks	3	(4.7)
Does not purchase drinks	23	(35.9)
Household types of water storage:		
Households that store water in buckets	63	(98.4)
Households that store water in bottles	10	(15.6)
Household reports running out of water for handwashing:		
“Never”	12	(18.8)
“Rarely”	27	(42.2)
“Sometimes”	25	(39.0)
Household perceives the use of chlorine in water as good for health		
Yes	52	(81.2)
No	12	(18.8)

* Mean (standard deviation); † clay container of approximately 250 cc.

**Table 3:** The description of practices related to water, hygiene, and sanitation, in the two Shawi communities, Peru (*n* = 64).

Practices Related to Water Quality	*n*	(%)
Water treatment practices:		
Boils or heats water	38	(59.4)
Filters water	0	(0.0)
Chlorinates water	0	(0.0)
Buys water	0	(0.0)
Allows the water to stand/sedimentation	36	(56.3)
Adds plants to water	2	(3.1)
Practices related to hygiene	*n*	(%)
Handwashing practices:		
Wash hands before preparing masato	42	(65.6)
Wash hands before preparing meals	44	(68.8)
Wash hands before eating	61	(95.3)
Wash hands after eating	47	(73.4)
Wash hands after defecating	23	(35.9)
Handwashing with soap:		
No	17	(26.6)
Only when soap is available	38	(59.4)
Yes	9	(14.0)
Practices related to sanitation	*n*	(%)
Practices open defecation	64	(100.0)
Open defecation site:		
Anywhere in the community	32	(50.8)
Spaces shared by family	24	(38.1)
Spaces shared with other families	7	(11.1)
